# An automated framework for evaluation of deep learning models for splice site predictions

**DOI:** 10.1038/s41598-023-34795-4

**Published:** 2023-06-23

**Authors:** Amin Zabardast, Elif Güney Tamer, Yeşim Aydın Son, Arif Yılmaz

**Affiliations:** 1 Department of Health Informatics, Graduate School of Informatics, Middle East Technical University, Ankara, Turkey; 2 Institute of Data Science, Maastricht University, Maastricht, The Netherlands

**Keywords:** Transcription, Transcriptomics, Data mining, Machine learning, Computational biology and bioinformatics, Molecular biology

## Abstract

A novel framework for the automated evaluation of various deep learning-based splice site detectors is presented. The framework eliminates time-consuming development and experimenting activities for different codebases, architectures, and configurations to obtain the best models for a given RNA splice site dataset. RNA splicing is a cellular process in which pre-mRNAs are processed into mature mRNAs and used to produce multiple mRNA transcripts from a single gene sequence. Since the advancement of sequencing technologies, many splice site variants have been identified and associated with the diseases. So, RNA splice site prediction is essential for gene finding, genome annotation, disease-causing variants, and identification of potential biomarkers. Recently, deep learning models performed highly accurately for classifying genomic signals. Convolutional Neural Network (CNN), Long Short-Term Memory (LSTM) and its bidirectional version (BLSTM), Gated Recurrent Unit (GRU), and its bidirectional version (BGRU) are promising models. During genomic data analysis, CNN’s locality feature helps where each nucleotide correlates with other bases in its vicinity. In contrast, BLSTM can be trained bidirectionally, allowing sequential data to be processed from forward and reverse directions. Therefore, it can process 1-D encoded genomic data effectively. Even though both methods have been used in the literature, a performance comparison was missing. To compare selected models under similar conditions, we have created a blueprint for a series of networks with five different levels. As a case study, we compared CNN and BLSTM models’ learning capabilities as building blocks for RNA splice site prediction in two different datasets. Overall, CNN performed better with $92\%$ accuracy ($6\%$ improvement), $89\%$ F1 score ($8\%$ improvement), and $96\%$ AUC-PR ($4\%$ improvement) in human splice site prediction. Likewise, an outperforming performance with $96\%$ accuracy ($11\%$ improvement), $94\%$ F1 score ($16\%$ improvement), and $99\%$ AUC-PR ($7\%$ improvement) is achieved in *C. elegans* splice site prediction. Overall, our results showed that CNN learns faster than BLSTM and BGRU. Moreover, CNN performs better at extracting sequence patterns than BLSTM and BGRU. To our knowledge, no other framework is developed explicitly for evaluating splice detection models to decide the best possible model in an automated manner. So, the proposed framework and the blueprint would help selecting different deep learning models, such as CNN vs. BLSTM and BGRU, for splice site analysis or similar classification tasks and in different problems.

## Introduction

The human genome annotation efforts benefit from the recent advances in RNA sequencing and transcriptomics studies, while splice site detection has become a significant research question. However, there is no guideline for selecting the best model for this task. Here we present a novel framework for automated evaluation of various deep learning-based splice site detectors. The framework eliminates time-consuming development by providing automated experiments for different models, architectures, and configurations to obtain the best model for a given RNA splice site dataset. Identification of the precise location is a critical challenge in human genome annotations. Therefore, determining the exon-intron boundaries of the genes is essential for identifying a gene structure. Splice sites determine the exon-intron and intron-exon boundaries that regulate RNA splicing, a post-translational modification process that converts pre-mRNA molecules to mature mRNAs.

**Figure 1:**
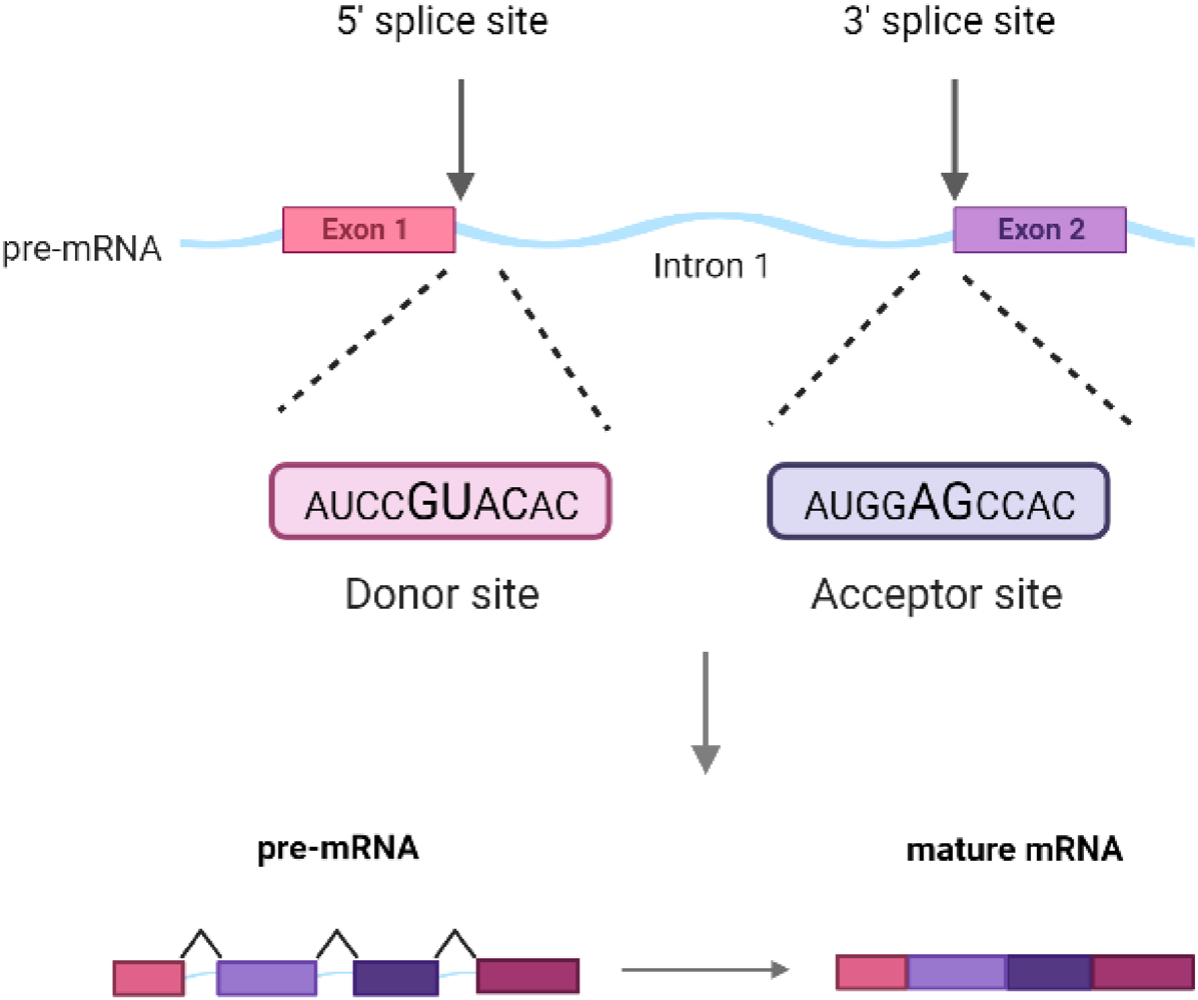
Splice sites are four conserved nucleotides: the donor sequence GU at the 5′ and the acceptor sequence AG at the 3′ end of an intron. After the splicing process pre-mRNA is converted to mature mRNA.

Also, alternative mRNAs can be obtained from the same gene sequence through the process known as alternative splicing. So, correct splice site recognition is critical for proper protein structure formation. Splice sites are typically composed of four conserved nucleotides: the donor sequence GT (GU for pre-mRNA) at the 5′ (at the exon-intron boundaries) and the acceptor sequence AG at the 3′ end (at the intron-exon boundaries) as in Fig. 1^1^. The splice sites that contain GT-AG sequences are called canonical splice sites. Likewise, splice sites do not contain GT-AG dimers called non-canonical splice sites.

Mutations in splice regions are linked with a variety of diseases. Most of these mutations are single nucleotide substitutions with consequences ranging from complete exon skipping to a nearby pseudo 3′ or 5′ splice site or conservation of the mutated intron. Moreover, mutations can introduce new splice sites within an exon or intron. Frasier syndrome, Myotonic dystrophy, Retinitis pigmentosa, and Spinal muscular atrophy are just a few of the many diseases caused by splice site mutations^2–4^. Several sequencing-based methodologies for identifying splice sites have been developed^5,6^. However, conducting wet lab studies to classify candidate splice site variants is not always feasible for clinical applications, so fast and accurate in-silico predictions of splice sites are needed. Many tools were developed for splice site prediction, but there is still no gold standard tool for clinical use to predict the effect of splice site variants. Therefore, in the literature, the methodologies need to be compared.

Many splice site annotation and prediction tools annotate and predict splice sites; many tools have been developed with different strategies, such as Fruit Fly Splice Predictor, Human Splicing Finder, RegRNA (A Regulatory RNA Motifs and Elements Finder), ESEfinder (Exonic Splicing Enhancers finder), GeneSplicer, and SpliceMachine. GeneSplicer uses the Decision Tree Algorithm with Markov models to train for signals around the splice sites^7^. SpliceMachine utilizes Support Vector Machines (SVMs) to solve this problem^8^. There are also other studies based on SVMs, artificial neural networks (ANN), and Random Forest (RF) algorithms for the identification and prediction of splice sites^9–11^. In addition, Bayesian networks^12^ and Maximum Entropy Distribution (MED) models^13^ are applied to the splice site prediction problem. In addition to these approaches, deep learning-based algorithms have also been applied to genome analysis.

Deep Learning algorithms, in general, are learning algorithms with an ensemble architecture that transforms data into various representations prior to classification/regression steps. Various deep learning algorithms are developed based on a central theoretical framework, which has effectively dealt with complex pattern recognition challenges in recent years. A deep neural network comprises several layers of perceptrons. A fully connected (dense) network input of a neuron in one layer is a linear combination of neuron outputs of the previous layers. A network that uses convolution operation to build layers is known as Convolutional Neural Network (CNN)^14^. CNN is beneficial when the data have some inherited local correlation. Deep Neural Networks, such as the Recurrent Neural Network (RNN)s, can be adapted to process long sequential data formats, where data is related to the prior information, and the neurons form a cycle. The output of a layer forms an input of the next layer, which allows the algorithm to have a theoretically infinite memory of the data sequence^15^. As a result, RNNs can take the sequential data as an input and generate sequential data as an output. RNNs provide a process of a more extended range of context information. However, there are some limitations of RNNs, such as the requirement of pre-segmented training data and vanishing gradient problems^16,17^. Long Short-Term Memory (LSTM) has successfully overcome these problems by allowing the constant unobstructed flow of error information between the input and output of each cell^18^. Bidirectional LSTM (BLSTM) based RNNs use forward and backward layers that allow access to a more extended range context in both directions^19,20^.

A local positive correlation exists in DNA/RNA sequences, and the sequence resembles a one-dimensional image. Convolutional layers are perfect for extracting information in such scenarios. Also, the strength of the relationship of bases in the genomic sequence is inversely related to their distances, rationalizing the use of CNNs^21^. In comparison, LSTMs are valid network structures for processing sequential data like text and time series. So, BLSTM utilizes the genomic data’s sequential nature. Since the DNA/RNA sequence can be interpreted from both directions and there is no difference between them, BLSTMs are used as a direction invariant model.

Several studies used CNN and RNN to analyze the genetic data patterns in recent literature. Jaganathan et al. have used ResNet^22^ like structures, named SpliceAI, to analyze Genome sequences as large as 10,000 nucleotide bases^23^. They have achieved a top-k accuracy of 95% using GENCODE^24^ data for training and validation. Zhang et al. have used simple CNNs, named DeepSplice, to analyze the GENCODE^24^ data for splice variant detection with 96.1% accuracy^25^. Another simple CNN approach proposed by Zuallaert et al. (SpliceRover) has also achieved up to 96% accuracy on different datasets of prior research^26^. Wang et al. have also used a CNN-based method (SpliceFinder) for predicting splice sites using the Ensembl genome database project’s^27^ data^28^. Splice2Deep is another CNN based-approach using the Ensembl genome database^29^. As an example of Bidirectional RNN-based approaches, Sarkar et al. have used different RNN-based networks, such as vanilla RNN, LSTM, and Gated Recurrent Unit (GRU) structures, to analyze NCBI’s Genbank data^30^ and achieved 99.95% accuracy^31^. Dutta et al. have used an RNN-based approach, specifically BLSTM, to predict splice junctions on a dataset generated from GENCODE annotations^32^. Both CNN and BLSTM-based networks can be used successfully to analyze genomic data^33^. Researchers have also tried a combination of CNNs and Bidirectional RNNs (henceforth referred to as “Hybrid Methods”. For example Alam et al. have tried a hybrid approach by combining CNNs and BLSTMs^34^. They reported their utmost accuracy as 98.8% on the HS3D dataset^35^. Also, CNN and BLSTM hybrid method has been shown to outperform CNN on the HS3D dataset^36^.

Various approaches with seemingly different architectures, as summarized in Table 2, do yield significant and highly accurate classification results on the different datasets for the prediction of splice sites. Overall, these results show that it is possible to classify splice sites using a deep neural network successfully. However, generalization of those performance results to all models is difficult; First, all the different layers of a Deep Neural Network are responsible for the regularization effects. However, when two architectures are deep and their inner structures are different, it is difficult to isolate the contribution of a specific part of each network. Additionally, both CNN and BLSTM model-based approaches with convolutional layers and Bidirectional LSTM cells are used in genomic studies and bioinformatics, but the principles for deciding the best approach based on the dataset’s internal structure are not clear.

Considering previous CNN and BLSTM-based splice site prediction models, in this study, we aim to compare these two promising networks’ performances to answer which Deep Neural Network approach is a better fit for the splice site prediction and similar problems. To our knowledge, no comprehensive comparison of BLSTM and CNN in splice site detection for various configurations has been reported. Therefore, there was a need to compare two different deep learning-based methods using standard datasets. Consequently, we designed a comparative experiment to aid in developing custom deep learning architectures based on CNN, BLSTM, or BGRU.

## Methodology

The novel framework for the automated evaluation of various deep learning-based splice site detectors eliminates time-consuming development and experimenting activities for different codebases, architectures, and configurations to obtain the best models for a given RNA splice site dataset. Therefore, it facilitates using the best models for the researchers working on RNA splicing site analysis.

**Figure 2:**
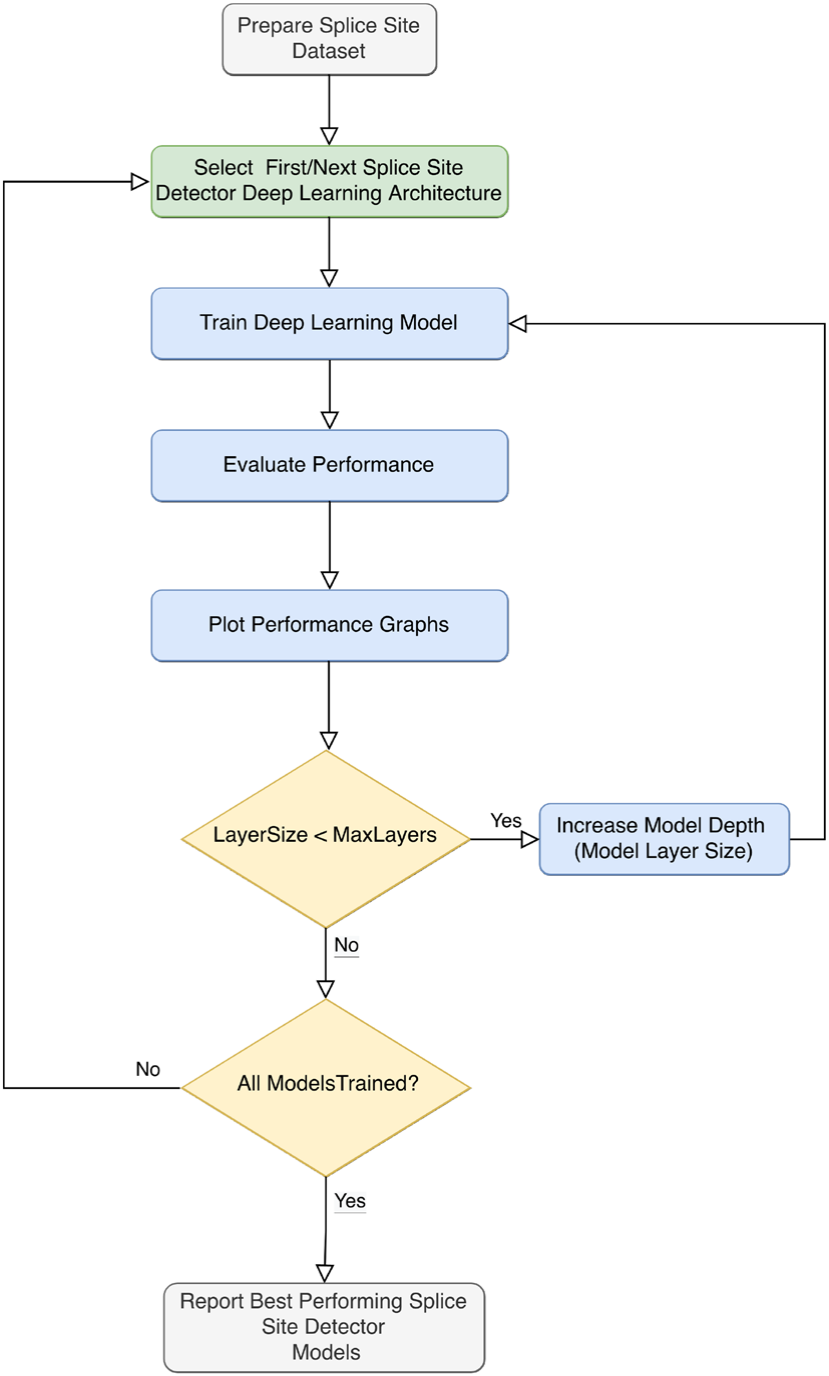
Automated deep learning model evaluation framework for splice site prediction. Selected Deep learning models CNN, LSTM, BLSTM, GRU or BGRU are trained for available datasets.

The framework operation is explained as a flowchart, as shown in Fig. 2. The framework can execute different deep learning architectures, such as CNNs, LSTMs, and GRUs, even if they are structurally different. Changing network depth from 1 to N in the framework is also possible. As seen on the flowchart, a network architecture is first selected. Then all experiments for various depth for the selected deep learning architecture is automatically performed. The resulting performance plots are automatically generated for each network for extensive evaluation. The set of experiments is repeated automatically for the following architecture. The process is finalized when experimenting with all deep architectures and models are finished.

The various network configurations are evaluated on the same datasets as in “Data” section. Convolutional and recurrent method performances are compared as representative deep learning approaches for the splice site prediction problem. Computation of these models may be explained with following mathematical expressions.

### Mathematical expressions for convolutional neural network (CNN) model

CNNs consists of convolutional layers which are characterized by an input map, a bank of filters and biases b. The output of a convolution layer with stride 1 and single convolution kernel is:

1 $$\begin{aligned} x_{i}^{l}=\sum _{m}w_{m}^{l} o_{i+m}^{l-1}+b_{i}^{l} \end{aligned}$$

Here, $o_{i}^{l}=f(x_{i}^{l})$: the output of any activation function, *l*: is the *l*th layer, *x*: is one dimensional input with dimension H, *w*: is the kernel with dimension *k* and iterator *m*, $w_{l}^{m}$: the weight vector connecting neurons of layer l with neurons of layer $l-1$, $b^{l}$: bias at layer *l*, $x_{i}^{l}$: the convolved input vector and kernel at layer *l* and bias, $o_{i}^{l}$: the output vector at layer *l*, *f*(.): the activation function, ReLU for all layers except last layer which uses softmax.

### Backpropagation and optimization

For backpropagation there are two updates performed, for the weights and for the gradients. In order to calculate the change for single weight parameter $w_{m^{\prime }}$, it is need to compute $\frac{\partial E}{\partial w_{m^{\prime }}^l}$. Error is calculated for *E* is error calculated with

In splice site prediction models, maximum likelihood estimation function is used for loss computation in training process of models. In training of models, the objective is to minimize the loss function. Gradient descent optimization was used in the framework to reduce the loss. The basic idea for gradient descent assumes that the loss functions are generally convex functions. If weights are updated in the opposite direction of the gradients, i.e. in descending direction, the weights are expected to reach the global minima. In back-propagation, the weights are updated by computing the gradient of loss function with respect to the output that needs to be back-propagated.

2 $$\begin{aligned}{} & {} L=-\tfrac{1}{N} \sum _{i=1}^{N}[o_{i}log(\hat{o}_{i})+(1-o_{i})log(1-\hat{o}_{i})] \end{aligned}$$

3 $$\begin{aligned}{} & {} \quad \delta _{i}^{l}={\frac{\partial E}{\partial x_{i}^{l}}} \end{aligned}$$

4 $$\begin{aligned}{} & {} \quad \frac{\partial E}{\partial x_{i^{\prime }}^{l}}=\sum _{m=0}^{k_{1}-1}\delta _{i^{\prime }-m^{l+1}w_{m}^{l+1}f^{\prime }\left( x_{i^{\prime }}^{l}\right) } \end{aligned}$$

5 $$\begin{aligned}{} & {} \quad \frac{\partial E}{\partial w_{m^{\prime }}^{l}}=\sum _{i=0}^{H-k_{1}}\delta _{i }^{l}o_{i+m^{\prime }}^{l-1} \end{aligned}$$

Similarly recurrent networks are trained using LSTM and GRU models. The LSTM model paremeters are computed as follows follows. An LSTM consists of input gate, forget gate and output gate.

### Mathematical expressions for Long Short-Term Memory (LSTM) model

A standard LSTM unit is composed of a cell, an input gate, an output gate and a forget gate. The cell stores values for arbitrary time intervals, and the three gates control the flow of information into and out of the cell. Forget gates decide what information to discard from a prior state by assigning a previous state, compared to a current input, a value between 0 and 1. A (rounded) value of 1 indicates that the information should be kept, whereas a value of 0 indicates that it should be discarded. Using the same approach as forget gates, input gates decide which pieces of new information to store in the existing state. The LSTM network can sustain useful long-term dependencies by selectively outputting appropriate information from the current state.

The input gate function shown in Eq. (6). is used to evaluate the importance of new information carried by the input.:

6 $$\begin{aligned} i_{t}=\sigma (w_{i}[h_{t-1},x_{t}]+b_{i}) \end{aligned}$$

Forget gate function in shon Eq. (7). is used to decide whether to keep the information from the previous time step or forget it:

7 $$\begin{aligned} f_{t}=\sigma (w_{f}[h_{t-1},x_{t}]+b_{f}) \end{aligned}$$

Similarly output gate function is shown in Eq. (8):

8 $$\begin{aligned} o_{t}=\sigma (w_{o}[h_{t-1},x_{t}]+b_{o}) \end{aligned}$$

LSTM model input cell input activation vector is computed using:

9 $$\begin{aligned} \tilde{c}_{t}=tanh(w_{c}[h_{t-1},x_{t}]+b_{c}) \end{aligned}$$

LSTM cell state vector is computed using:

10 $$\begin{aligned} c_{t}=f_{t}*c_{t-1}+i_{t}*{\tilde{c}}_{t} \end{aligned}$$

LSTM hidden state vector also known as output vector of the LSTM unit:

11 $$\begin{aligned} h_{t}=o_{t}*tanh(c^{t}) \end{aligned}$$

In the equations above, the terms may be explained as: $$x_t$$: input vector to the LSTM unit, $$f_t$$: forget gate’s activation vector, $$i_t$$: input/update gate’s activation vector, $$o_t$$: output gate’s activation vector, $$h_t$$: hidden state vector also known as output vector of the LSTM unit, $$\tilde{c}_{t}$$: cell input activation vector, $${c}_{t}$$: cell state vector, $$w_i,w_f,w_o, $$: weights, $$b_i,b_f,b_o, $$: biases.

### Mathematical expressions for Gated Recurrent Unit (GRU) model

The GRU is similar to an LSTM with a forget gate, but it has fewer parameters than an LSTM because it does not have an output gate. Because to their comparable designs and often similarly performance, GRU and LSTM can both be seen as variations of each other. GRU employs update and reset gates to tackle the vanishing gradient problem of a regular RNN. Essentially, there are two vectors that determine what information should be transmitted to the output. They are unique in that they can be trained to retain knowledge from a long time ago without being washed away by time or to discard information that is unnecessary to the prediction.

The update gate function shown in Eq. (12) enables the model to determine how much past knowledge (from earlier time steps) must be passed on to the future.

12 $$\begin{aligned} z_{t}=\sigma (w_{z}[h_{t-1},x_{t}]+b_{z}) \end{aligned}$$

The model’s reset gate is used to determine how much of the past knowledge to forget is shown in Eq. (13):

13 $$\begin{aligned} r_{t}=\sigma (w_{r}[h_{t-1},x_{t}]+b_{r}) \end{aligned}$$

Here, GRU candidate activation vector is computed as follows:

14 $$\begin{aligned} \tilde{h}_{t}=tanh(w_{h}[x_t,r_{t}*h_{t-1}]+b_{c}) \end{aligned}$$

Then, GRU output vector:

15 $$\begin{aligned} h_{t}=r_{t}*h_{t-1}+(1-z_{t})*{\tilde{h}}_{t} \end{aligned}$$

In the equations above the terms may be explained as: $$x_t$$: input vector to the GRU unit, $$f_t$$: forget gate’s activation vector, $$i_t$$: input/update gate’s activation vector, $$o_t$$: output gate’s activation vector, $$h_t$$: hidden state vector also known as output vector of the LSTM unit, $$\tilde{c}_{t}$$: cell input activation vector, $${c}_{t}$$: cell state vector, $$w_i,w_f,w_o $$: weight matrices, $$b_i,b_f,b_o $$: biases.

### Mathematical expressions for BLSTM and BGRU models

BLSTM and BGRU models are bidirectional versions of consists of LSTM and GRU cells as in unidirectional models. However, they one more LSTM layer, namely forward and backward layers to read the input sequence which reverses the direction of information flow. This means that the input sequence flows backward in the additional LSTM layer. Then the outputs of forward and backward layers are combined from both forward and backward layers by averaging.

We applied the following principles to ensure that the specific differences were reduced and that the network designs were comparable:

1. The experiments are separated into multiple groups (based on the family of the network) with multiple levels (based on the complexity of the network within the same family). Each level is directly comparable to its counterpart from the other family. Multiple levels in the same family group are comparable on the grounds of complexity.
2. Smaller networks are preferred to lower the possibility of deviation between two groups, which is expected to be higher if broader (and deeper) networks are used.
3. The amount of network’s trainable parameters for the same levels should be approximately the same between two groups of families (Table 1). Since the learning capacity is directly proportional to the number of trainable parameters, we can make the networks more comparable by keeping the number of trainable parameters and their growth rate similar in each network.
4. Neural networks are created from many components, each of which has a role in regularizing the network. The reusable parts of the two families’ networks are kept the same to control the architecture.

Networks in each group are structurally similar but different in their design. A summary of the number of trainable parameters for each experimental setup is presented in Table 1.

**Table 1:** Number of trainable parameters in different networks with different layers.

	5 Layers	4 Layers	3 Layers	2 Layers	1 Layer
BLSTM	993	871	759	647	535
BGRU	891	795	699	603	507
CNN	683	631	579	527	475

**Figure 3:**
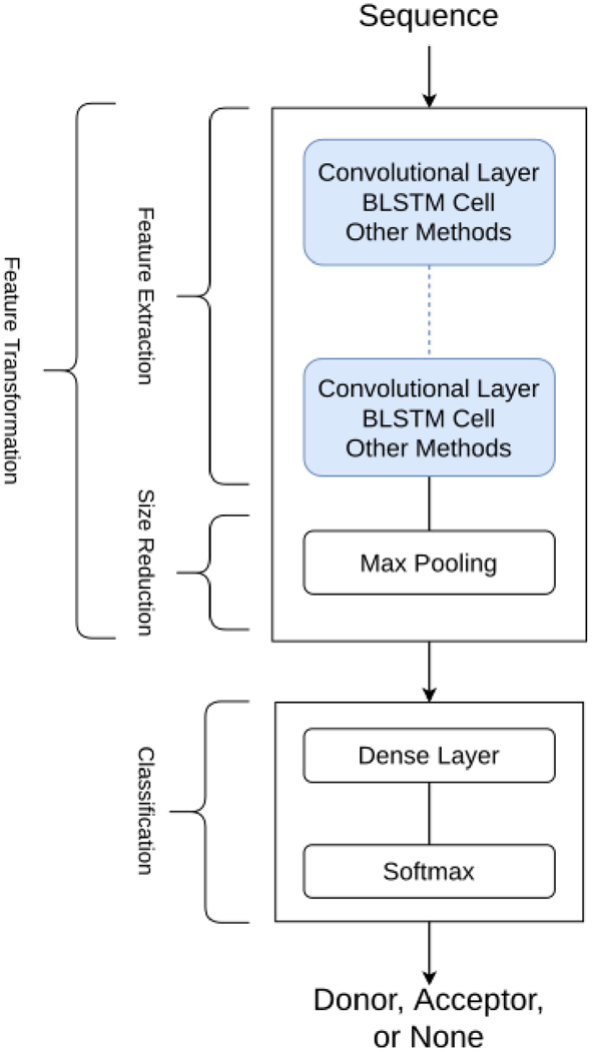
Framework allows processing of blueprints for different network architecture groups. This blueprint allows the comparison of networks with convolutional layers or recurrent cells such as BLSTM, which can also be used with other compatible methods.

The finalized framework of the proposed blueprint is presented in Fig. 3, and the details are explained in the “Results” section. These networks included a maxpooling layer to limit the number of trainable variables. In addition, we used Stochastic Gradient Descent (SGD) for the optimization method, and the loss function is cross-entropy.

### Model selection criteria

To compare the performances of various deep learning architectures, we identified the most frequently used architectures as CNN, BLSTM, and BGRU, which are reviewed in Tables 2 and 3. Therefore we focused our experiments on these models. Additionally, as Sarkar et al. used GRU and achieved good performance^31^, we included GRU and LSTM in the experimental models.

**Table 2:** Overview of the deep learning-based methods for splice site detection available in the literature: (D) Donor, (A) Acceptor, Acc. (Accuracy), Sn. (Sensitivity), Sp. Specificity.

Reference	Base	Architecture	Data	Sequence numbers/lengths		Measure	Performance	
DeepSplice^43^	CNN	Input layer 2 Conv layers 1 Dense layer Output layer	GENCODE	291,030 (true) 271,937 (false)	120 nt		**Donor**	**Acceptor**
						**Acc.**	0.907	0.893
						**Sn.**	0.917	0.873
						**Sp.**	0.897	0.913
SpliceRover^26^	CNN	Input layer 2 conv layers+ Max pooling layer 1 Dense layer Output layer	NN269	1324 (true) (D) 4922 (false)(D) 1324 (true) (A) 5553 (false)(A)	15 nt(D) 90 nt(A)	**Acc.**	0.9535	0.9612
						**Sn.**	0.9011	0.9077
						**Sp.**	0.9674	0.9739
						**auPRC**	0.9829	0.9899
SpliceFinder^28^	CNN	Input layer A conv layer Dense layer Output layer	Ensembl (hg38 dataset)	30,000^b^	40–400 nt	**Acc.**	0.969–0.832 (40 nt) 0.965–0.903 (400 nt)	0.969–0.832 (40 nt) 0.965–0.903 (400 nt)
DeepSS^44^	CNN	Input layer 2 Conv layers+ Max pooling layer 2 dense layer Output layer	CE	750 (true)(D) 19,250(false)(D) 1000 (true)(A) 19,000 (false)(A)	141 nt	**Acc.**	0.97^a^	0.96^a^
						**Sn.**	0.95^a^	0.93^a^
						**Sp.**	0.97^a^	0.96^a^
						**Pr.**	0.87^a^	0.83^a^
						**MCC**	0.89^a^	0.85^a^
						**AUC ROC**	99.47^a^	99.56^a^
						**AUC PR**	97.88^a^	98.18^a^
DeepSS^44^	CNN	Input layer 2 Conv layers+ Max pooling layer 2 dense layer Output layer	NN269	1324(true)(D) 4922(false)(D) 1324(true)(A) 5553(false)(A)	15 nt(D) 90 nt(A)	**Acc.**	0.93^a^	0.97^a^
						**Sn.**	0.91^a^	0.93^a^
						**Sp.**	0.96^a^	0.97^a^
						**Pr.**	0.86^a^	0.9^a^
						**MCC**	0.85^a^	0.9^a^
						**AUC ROC**	98.43^a^	99.34^a^
						**AUC PR**	93.97^a^	97.32^a^
Splice2Deep^29^	CNN	Input layer Conv. layer Max pooling layer Output layer	Ensembl (hg38 dataset)	250,400 (true) 250,400 (false)	602 nt	**Acc.**	97.38	96.91
						**Sn.**	95.93	95.61
						**Sp.**	98.83	97.8
						**F1 Score**	96.38	96.91
						**AUC**	99.1	98.69
Sarkar et. al.^31^	Vanilla RNN, LSTM, GRU	3 stacks (90 Vanilla RNN, GRU or LSTM cells)	GenBank (splice-junction gene sequences)	3175^b^	60 nt	**Acc.**	1.00	99.95
						**Sn.**	1.00	1.00
						**Sp.**	1.00	99.93
						**F1 Score**	1.00	99.93
Splice AI^23^	ResNet	Input layer Conv. layer Residual blocks (Batch-normalization layers Rectified linear units Convolutional layers) Output layer	GENCODE	13,796 donor–acceptor pairs	40–5000 nt		Combined donor and acceptor	
						**Acc.**	0.95	
						**AUC PR**	0.98	

^a^The approximate values were taken from the graphs since exact values were not given in the paper. Only imbalanced dataset results were mentioned for DeepSS.

^b^The number of true and false splice site sequences was not mentioned.

**Table 3:** Summary of studies with deep learning based methods for splice site detection with the HS3D dataset. (D) Donor, (A) Acceptor, Acc. (Accuracy), Sn. (Sensitivity), Sp. Specificity.

Reference	Base	Architecture	Data	Sequence numbers/lengths		Measure	Performance	
DeepSplice^43^	CNN	Input layer 2 Conv layers 1 dense layer Output layer	HS3D	2796 (true)(D) 271937 (false)(D) 2880 (true)(A) 329,374 (false)(A)	140 nt		**Donor**	**Acceptor**
						**Acc.**	0.946	0.923
						**Sn.**	0.957	0.934
						**Sp.**	0.938	0.914
DeepSS^44^	CNN	Input layer 2 Conv layers + max pooling layer 2 dense layer Output layer	HS3D	2796 (true)(D) 90,953 (false)(D) 2880 (true)(A) 90,353 (false)(A)	140 nt	**Acc.**	0.97^a^	0.98^a^
						**Sn.**	0.96^a^	0.97^a^
						**Sp.**	0.97^a^	0.98^a^
						**Pr.**	0.88^a^	0.92^a^
						**MCC**	0.90^a^	0.93^a^
						**AUC ROC**	99.02	98.79
						**AUC PR**	95.93	94.28
DeepDSSR^34^	Hybrid (CNN + BLSTM)	2 inception like layers A convolutional layer A bidirectional layer Dense layer	HS3D	2796 (true)(D) 90,924 (false)(D)	140 nt	**Sn.**	0.988	–
						**Sp.**	0.891	–
						**MCC**	0.914	–

^a^The approximate values were taken from the graphs since exact values were not given in the paper. Only imbalanced dataset results were mentioned for DeepSS.

Also, these architectures are a good fit for the characteristics of genomic data. Firstly, there is a local relationship between a base and other bases in its vicinity in genomic data. A CNN architecture effectively interprets these local relationships^37^. Secondly, genomic data is sequential and recursive architectures—such as BLSTMs—are effective in interpreting sequential data^38^.

Genomic sequences can be analyzed better if they are inspected forward and reverse directions. The use of unidirectional networks may cause the loss of valuable information. In order to validate this expectation, we also experimented with unidirectional networks. Results of unidirectional and bidirectional versions of GRU and LSTM architectures are presented in “Results” section.

### Data

In evaluating our framework, we experimented with two splice prediction datasets, the HS3D and the *C. elegans*, where the details of the datasets are as follows.

#### HS3D dataset

We used the HS3D dataset in our experimental design^35^. This dataset includes 609,909 140 base pair(bp) long sequences located around splice sites. In true class, the splice site is located precisely in the DNA sequence’s middle on the 70th and 71st bps including only canonical GT-AG motifs. The false class was created by selecting the GT-AG pairs in not splicing locations. The false sites are located in range of ± 60 distance from true splice site location. The dataset may be downloaded using the script available in the GitHub repository using the link in Data availability section.

The HS3D dataset is publicly available and well-defined in false and true splice site sequences. The HS3D dataset is selected since it was successfully used in the CNN and BLSTM-based neural network approaches for splice site recognition, as listed in (Table 3) with the performance measures of each study. Moreover, two additional studies used the BLSTM and the CNN hybrid approach using the HS3D data to predict splice sites^34,36^. The HS3D is selected as a suitable benchmark dataset for comparing selected networks based on these observations.

During the preprocessing, the DNA sequences coded with IUPAC nomenclature (A, C, G, and T) are converted to a vector of length 4 (One-Hot Encoding), which is a compatible format for neural network studies^39^. All sequences in the HS3D dataset are categorized into four classes: true donor or acceptor splice sites and false donor or acceptor splice sites. Succeeding the literature, which split the data into a true donor, true acceptor, and non-site^28,40^ false groups are combined. So, we combined the false donor and acceptor groups, and after preprocessing, our final dataset had three classes: true donor, true acceptor, and non-site.

There were 2796 sequences in the true donor class and 2880 sequences in the true acceptor class; therefore, the true donor and true acceptor classes were approximately balanced. However, a high number of sequences belonging to the none-site class were in the dataset, with a count of 604,233. The large number of false sequences was the leading cause of the unbalanced classes. We balanced the dataset by downsampling the majority class (non-site) in a quasirandom manner. Thus, all classes were balanced and approximately had the same number of sequences after downsampling.

#### *C. elegans* dataset

The second dataset we used in our experiments is the *C. elegans* dataset, which is publicly available^41^. The dataset is composed of 17,300 false donor/acceptor and true 6700 donor/acceptor splice sites.

*C. elegans* dataset included 141 bp long sequences located around splice sites. The canonical splice site is located on the 63rd and 64th base pairs in the donor dataset, and in the acceptor dataset, the canonical splice site is located on the 60th and 61st base pairs. False splice site sequences are obtained from intronic regions and centered around non-splice site AG dinucleotides and GT dinucleotides.

During the pre-processing, the DNA sequences coded with IUPAC nomenclature (A, C, G, and T) are converted to a vector of length 4 (One-Hot Encoding), a compatible format for neural network studies. Again, the false donor and acceptor groups are combined, so after pre-processing, our final dataset had three classes: true donor, true acceptor, and non-site. Also, since our network is trained for 140 bp long sequences, sequences are trimmed one base from the right site. After this step, the *C. elegans* dataset had 140 bp long sequences. Since the non-site class has a high number of sequences compared to true donor and acceptor sites, similar to HS3D dataset, we balanced the dataset by downsampling the majority class (non-site) in a quasirandom manner. Thus, all classes were balanced and approximately had the same number of sequences after downsampling.

### Analysis

Several groups of experiments are created for different neural networks. Each experimental group includes multiple networks with a specific neural network layer, such as CNN, BLSTM, or others. Networks in each group are structurally similar but different in their design. During training, tenfold cross-validation is performed to split the data before training each network. In general, cross-validation eliminates the possibility of overfitting due to misrepresentative data selection. Also, repetitive experimentation with cross-validation eliminates the effects of randomness introduced by initiating the variables within the network and mini-batches. Each network has been trained ten times for 300 epochs with additional training for the BLSTM networks. The BLSTM networks with 1000 epochs have “extended” as the prefix. The networks are created using TensorFlow 2.3.0, and the training is done using Nvidia RTX 2080 Ti GPU. Results of all experiments are fully reproducible and available at our GitHub repository, explained in Code availability section.

### The evaluation metrics

Classification performance for all models is evaluated using accuracy and F1 score measurements as evaluation metrics. The Area Under the Curve-Precision-Recall (AUC-PR) is also calculated since it uses all the aspects of the confusion matrix in its final score computation^42^. As we aim to compare the performance gain at each level and in-between types of networks, we compared the performance of each network family at progressive levels during the evaluation instead of the outcomes. We expect each network family to improve its evaluation metric as more layers are constructed for feature transformation. Since corresponding levels in each network are designed to be comparable, the group of networks with the most significant increase in performance resulting from any added layer is favored.

## Results

In this study, we implemented a novel framework for the automated evaluation of deep learning based splice site detectors for a given RNA splice site dataset. We extensively tested our framework with two different splice datasets namely HS3D and *C. elegans*. As a first task, we tested our framework to determine if there is any difference in performance of CNN and BLSTM architectures as building blocks of the network’s feature transformation structure.

In the first step, we tested our framework to determine if there is any difference in the performance of CNN and BLSTM architectures as building blocks of the network’s feature transformation structure with the HS3D dataset. Next, the best-performing configurations identified are applied during training with BLSTM and CNN models for the *C. elegans* dataset shown in Fig. 7. Later, we used the framework to evaluate other architectures for selected configurations such as LSTM, GRU, BGRU.

### The framework for evaluation of splice site detectors

We proposed a framework that evaluated deep learning networks intended to take a sequence of DNA nucleotides and return the probability of the sequence belonging to a class (classification problem). The proposed framework represented in Fig. 3 consists of networks that have four main parts:

1. The input data: The input data is a sequence of one-hot-encoded DNA nucleotide bases, in which the length of each sequence is 140 nucleotides.
2. Feature extraction layers: Cumulatively, these layers will transform the data from one space to another where the classification can be achievable. The network consists of multiple repeating layers, such as CNN layers or BLSTM cells.
3. Following the feature extraction layers, the output layer is a classifier, consisting of a Dense layer construct with a softmax as an activation function.
4. The output consists of three values, reporting the probability of belonging to a particular class.

### Performance analysis of models using HS3D data

Several experiments are designed and conducted with networks based on the proposed framework. Although the framework does not impose any limit, in the experiments, we limited the number of layers to up to five different levels in feature transformation blocks. We discovered that networks containing BLSTM cells require more epochs during the loss plots training to reach a plateau state, so these networks are trained for extended duration until 1000 epochs. Figure 5 shows our experiments for comparing BGRU and BLSTM architectures. As it may be seen there is minimal difference between their performance, but as mentioned before BLSTMs are the more prominent version in the literature. Figures 6 and 7 shows performance per epoch for a subset of the experiments with the HS3D dataset and *C. elegans* dataset, respectively. All the networks involved in experiments have reached a stable performance level after the training and learned general knowledge about the dataset and match performance in training and test. There was no divergence between the training and validation plots.

**Figure 4:**
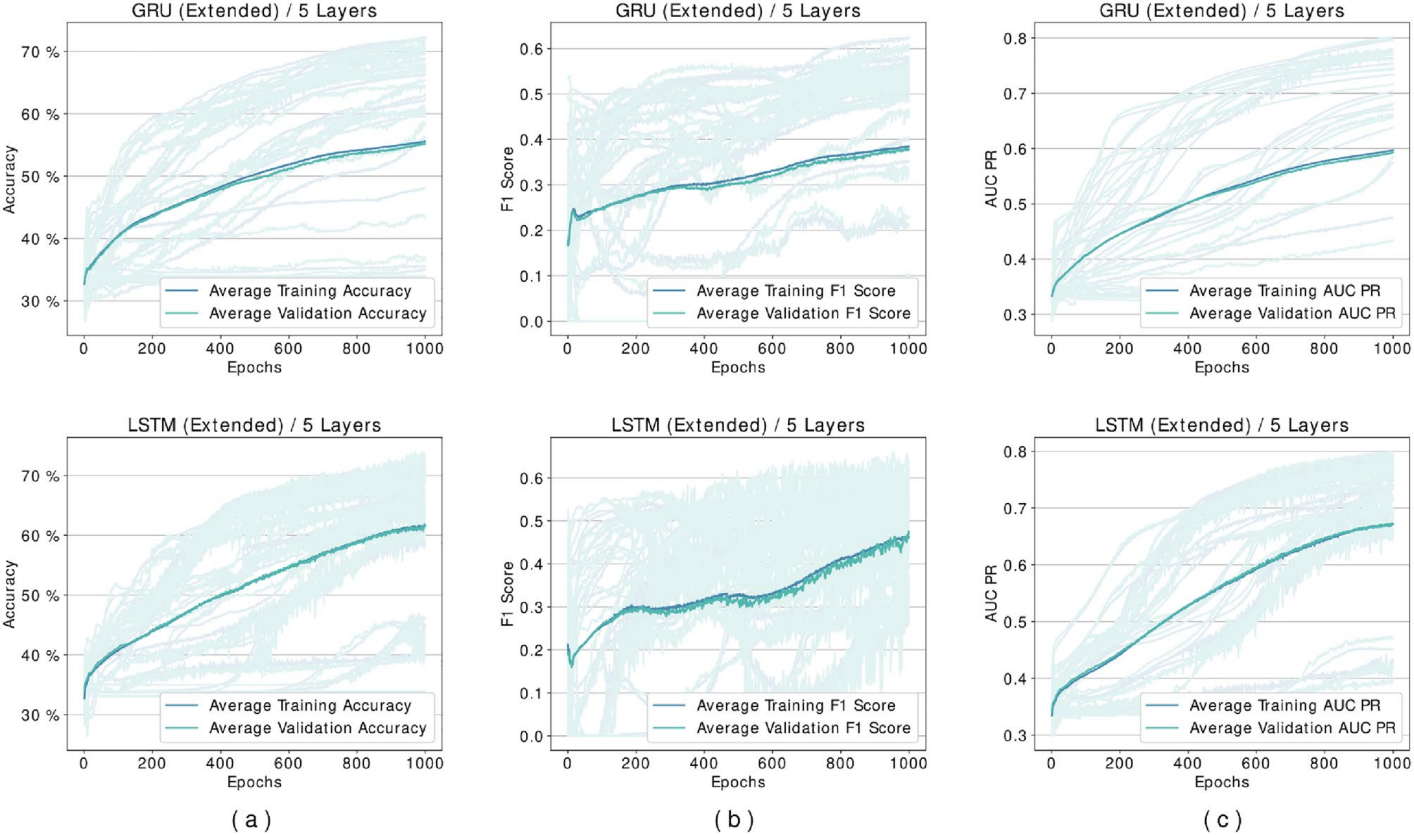
Comparison of GRU and LSTM architectures’ performance for HS3D dataset. These networks are performing suboptimally compared to their bidirectional counterparts, and they are also unable to learn any distinguishing features in some of the experiments. Columns from left to right are (**a**) Accuracy per epoch, (**b**) F1 Score per epoch, and (**c**) AUR-PR per epoch.

**Figure 5:**
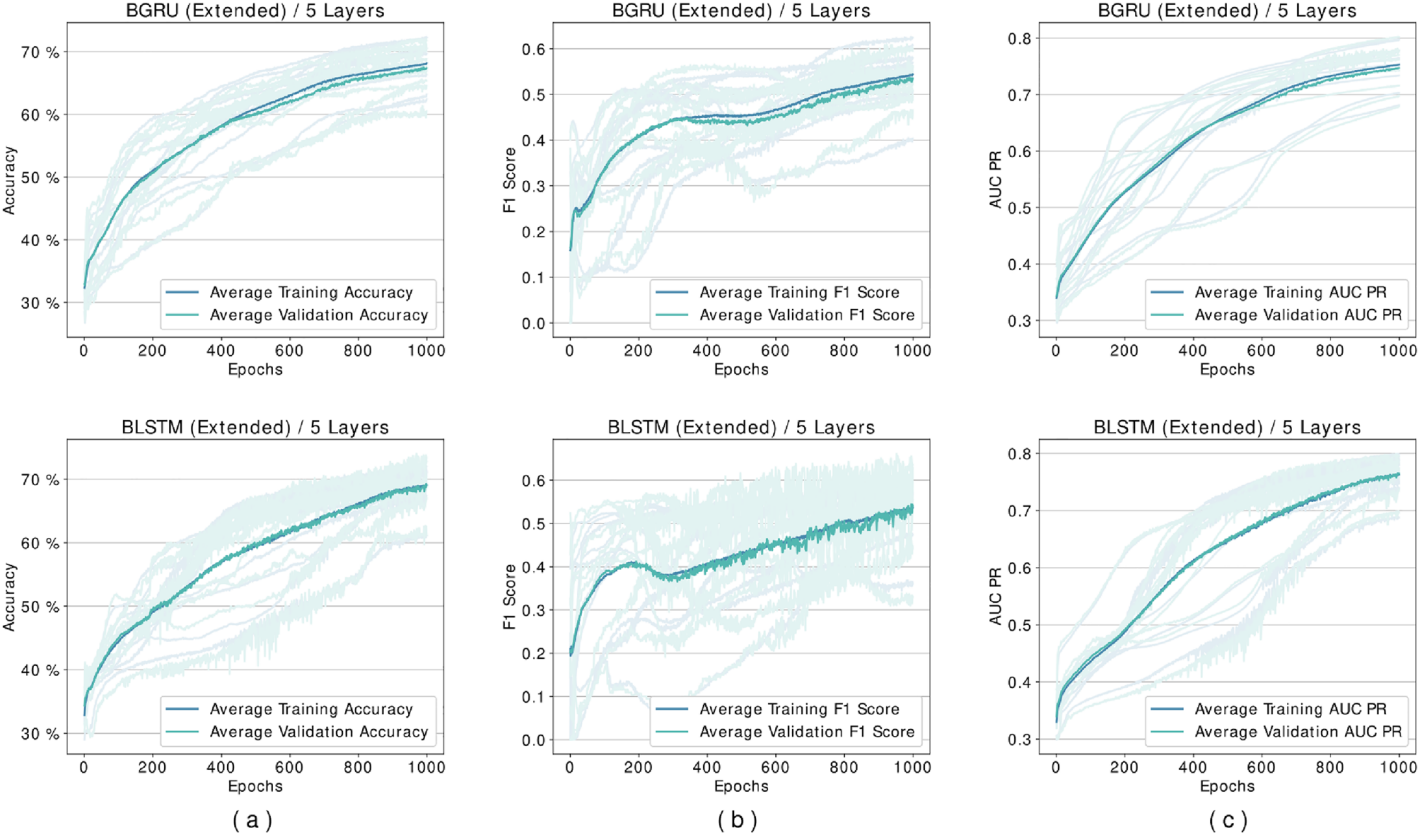
Comparison of BGRU and BLSTM architectures’ performance for HS3D dataset. There is no significant difference between the two architectures performances. Columns from left to right: (**a**) Accuracy per epoch, (**b**) F1 Score per epoch, and (**c**) AUR-PR per epoch.

**Figure 6:**
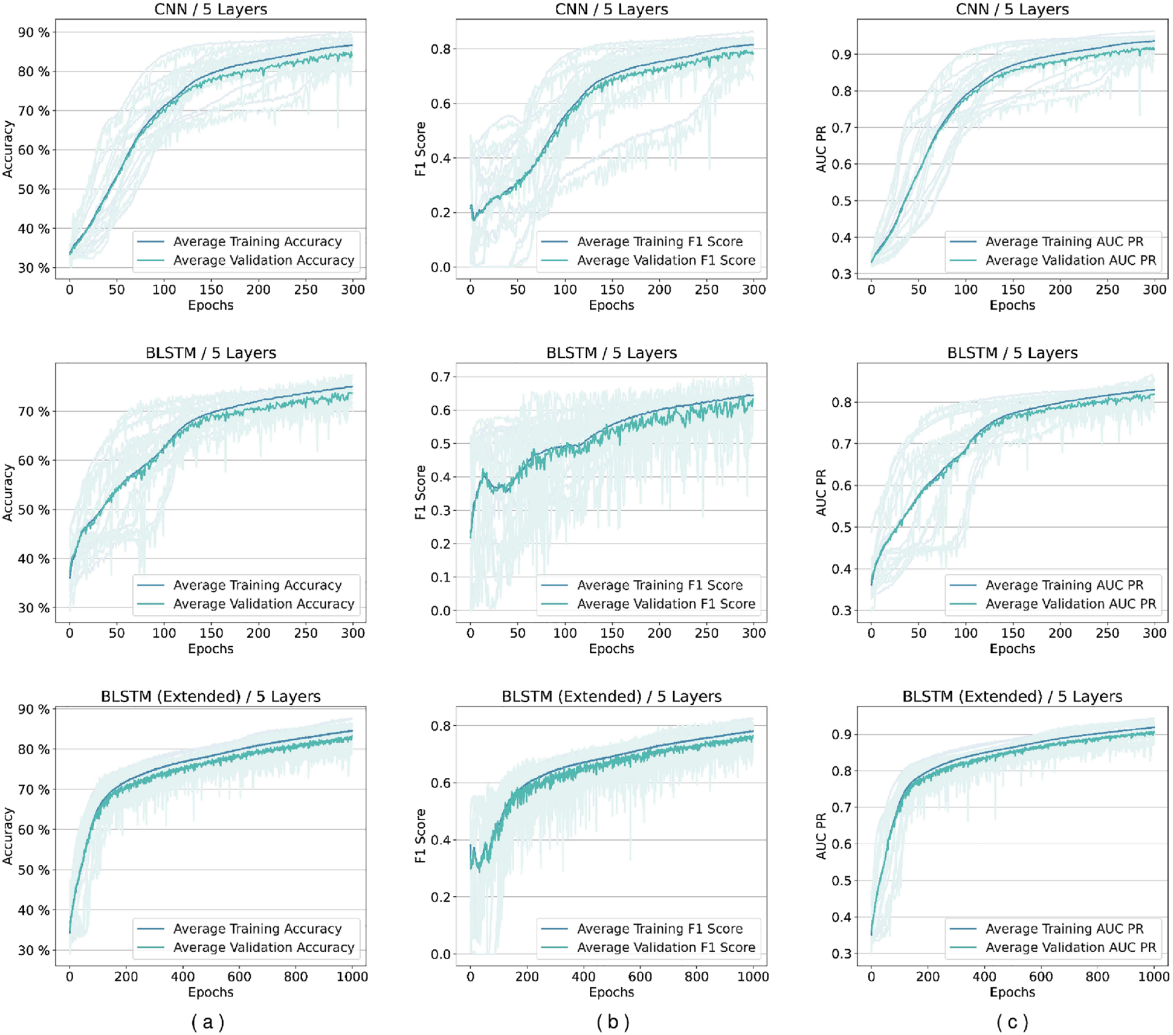
Performance per epoch for five-layer networks in HS3D dataset. Rows from top to bottom are for CNN, BLSTM, and extended BLSTM. Columns from left to right are (**a**) accuracy per epoch, (**b**) F1 score per epoch, and (**c**) A.U.C. Precision-Recall (PR), respectively. There is no indication of overfitting. All the training is completed successfully, and there is no gap between validation and training performance lines.

The best-performing model for CNN architecture (based on accuracy as the deciding measure) was obtained at a three-layer configuration for the HS3D dataset (Fig. 8a). Between one-layer and three-layers CNN networks trained, $6\%$ accuracy improvement was achieved, while extended BLSTM networks improved their accuracy by $5\%$ (Fig. 8a). Also, the CNN architecture achieved a maximum accuracy of $92\%$ compared to the base model and achieved a maximum score of $85\%$. In order to validate this expectation, we also experimented with unidirectional networks. Results of unidirectional and bidirectional versions of GRU and LSTM architectures are shown in Figs. 4, 5 and 6.

### Performance analysis of models using *C. elegans* data

**Figure 7:**
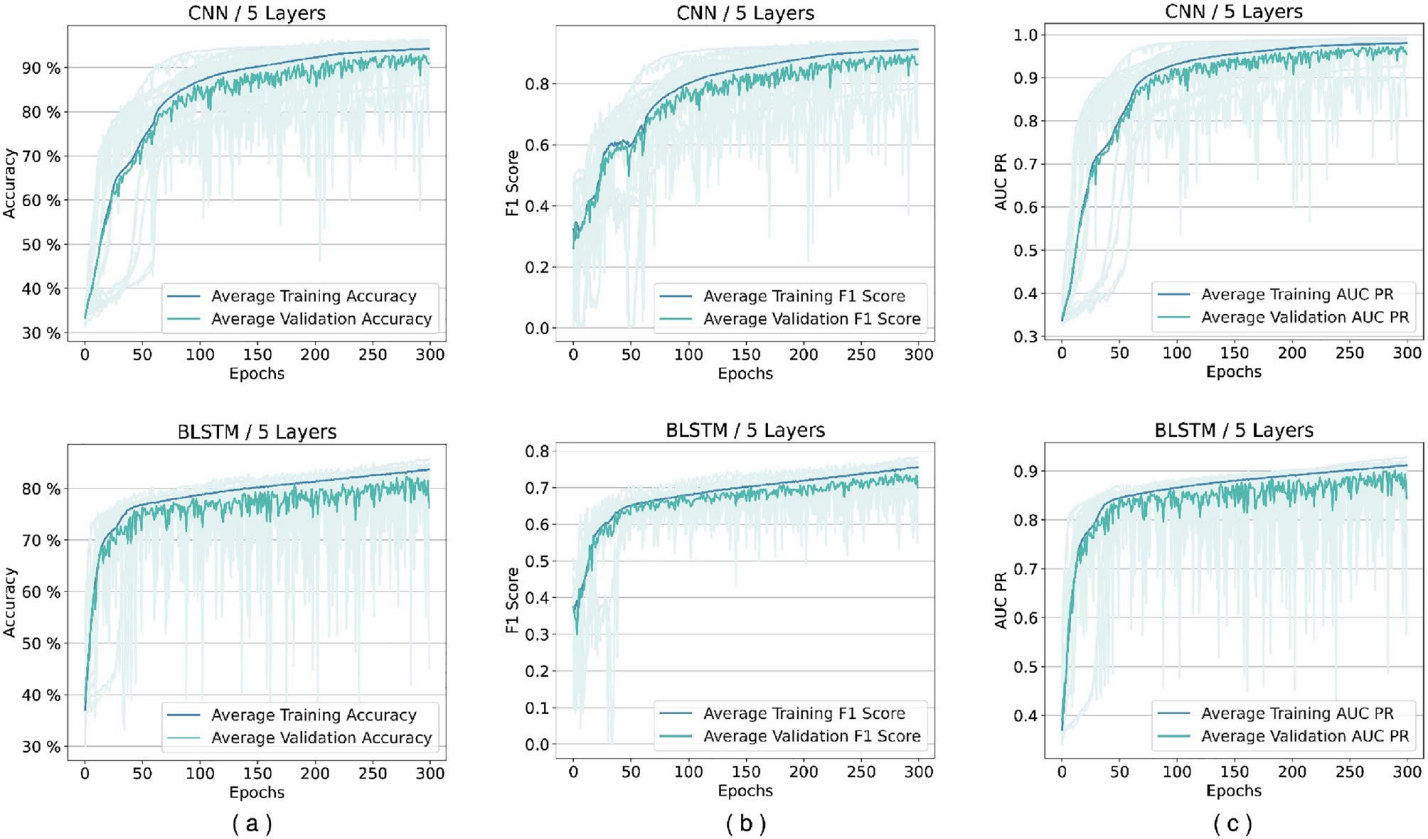
Performance per epoch example for five-layer networks in *C. elegans* dataset. Rows from top to bottom are, respectively, for CNN and BLSTM. Columns from left to right are (**a**) accuracy per epoch, (**b**) F1 score per epoch, and (**c**) A.U.C. Precision-Recall (PR), respectively. There is no indication of overfitting. All training was successful, and there is no gap between validation and training performance lines.

*C. elegans* dataset is used for the confirmation, and the results verify that CNN is the best-performing network architecture Fig. 7.

**Figure 8:**
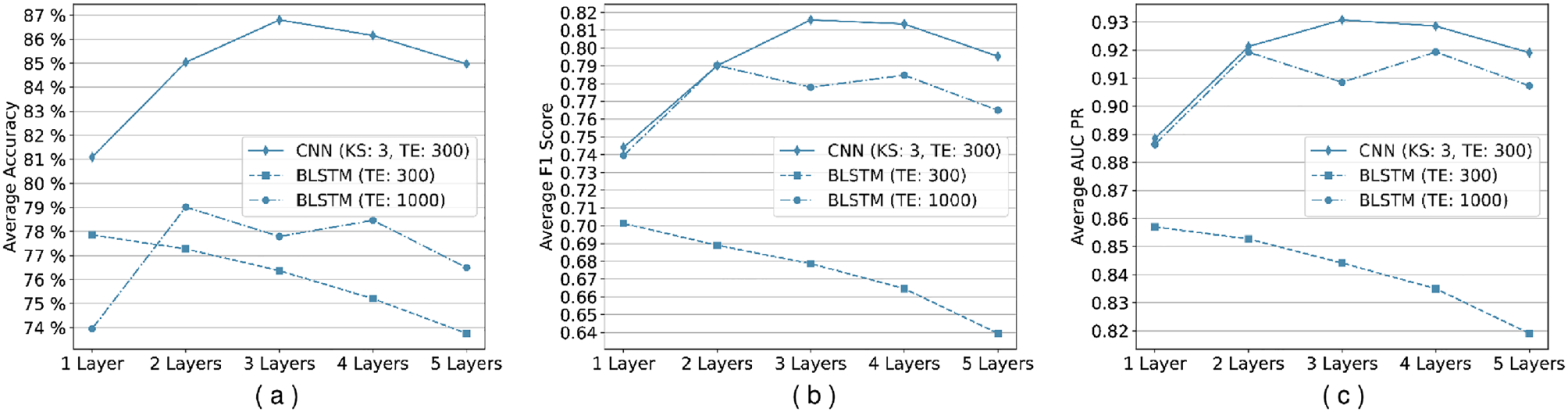
Comparison of the change in performance metrics types with each additional layer. We can conclude from all the metrics that adding convolutional layers improves the performance better than Bidirectional LSTM cells. From left to right, (**a**) average accuracy per epoch, (**b**) average F1 score per epoch, and (**c**) average A.U.C. Precision-Recall (PR) per epoch. KS: Kernel Size, TE: Termination Epoch.

Using the splice site prediction framework, we provided time required for training of models with respect different number of layers. Our results showed that CNN requires least time for training.

Also, we compared CNN and BLSTM models with the highest learning capacity for both HS3D and *C. elegans* datasets using the F1 score and AUC-PR metrics. The CNN architecture improved the F1 score by $8\%$ compared to the base model, and achieved a maximum score of $89\%$. The extended BLSTM improved the F1 score by $5\%$ and achieved a maximum of $85\%$ (Fig. 8b). Similarly, for the AUC-PR metric, CNN architecture improved its score by $4\%$ and achieved a maximum of $96\%$. The extended BLSTM improved its score by $3\%$ and achieved a maximum of $94\%$ (Fig. 8c).

**Figure 9:**
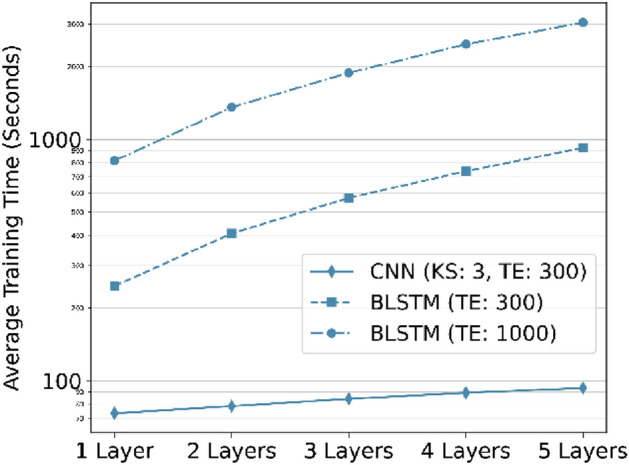
Comparison of training time for different network types with respect to additional layers (The Y axis is on a logarithmic scale). Convolutional networks are exponentially faster to train and use in comparison to BLSTM networks. KS: Kernel Size, TE: Termination Epoch.

Table 4 shows the results when framework is set to test all models for 5 layers for highest accuracy. It may be seen that CNN model performed best in accuracy and and F1 for HS3D dataset. Because, genomic data has learnable features in forward and reverse direction, bidirectional models (BLSTM and BGRU) performed better compared to unidirectional models (LSTM and GRU).

**Table 4:** Comparison of highest performance metrics of for various models.

Model (5 layers)	Dataset	Accuracy	F1	AUCPR
CNN	HS3D	**0.90**	**0.85**	**0.95**
LSTM (extented)	HS3D	0.66	0.56	0.74
GRU (extented)	HS3D	0.61	0.56	0.66
BLSTM	HS3D	0.77	0.71	0.87
BLSTM (extented)	HS3D	0.87	0.82	0.94
BGRU (extended)	HS3D	0.72	0.61	0.80
CNN	*C. elegans*	**0.96**	**0.94**	**0.99**
BLSTM	*C. elegans*	0.85	0.78	0.92

Significant values are in bold.

## Discussion

Selection of the best model for a machine learning task has become essential in Artificial Intelligence (AI) applications. The performance of different machine learning models may differ for a training dataset, which cannot be foreseen before the experiments. Here, we explained a novel framework for the automated evaluation of various deep learning-based splice site detectors. Our framework eliminates the laborious process of evaluating multiple models for selecting the best architecture and configuration for a given problem.

In this study, we have worked with an RNA splice site dataset; as splice site variants are associated with many diseases, identifying the splice site variants is critical. Mainly, the coding variants are considered disease-causing variants. However, non-coding variants with different consequences might affect the phenotype. To this extent, predicting which sequences are potential splice sites would help predict candidate variants with pathogenic outcomes, and prioritizing sequencing variants based on their effect on splicing aids in diagnosing genetic diseases.

Other researchers applied deep learning methods to splice site prediction, and different deep neural networks have been extensively studied in the literature without providing a generic approach. Both the CNN-based and the BLSTM-based deep neural networks can learn genomic data with significant accuracy. DeepSplice used a CNN-based network and evaluated human RNA-seq data obtained from GENCODE and HS3D datasets, which obtained an accuracy of around $90\%$^43^. SpliceRover used a CNN-based network, evaluated human NN269, and obtained an accuracy of around $90\%$^26^.DeepSS used a CNN-based network and evaluated *C. elegans* data, human NN269 data, and human HS3D data and obtained accuracy between $93$–$98\%$^44^. SpliceFinder used a CNN-based network, evaluated the human dataset downloaded from Ensembl, and obtained an accuracy of around $96\%$^28^. Splice2Deep used a CNN-based network and evaluated the human dataset downloaded from Ensembl and obtained accuracies of around $97\%$^29^. Unlike the previous studies, SpliceAI used different network architecture called Resnets and evaluated the human dataset downloaded from ENCODE, obtaining $95\%$ accuracy^23^.Besides these convolutional neural networks, there are BLSTM-based or hybrid studies. For instance, in one study, the BLSTM network was evaluated on the *C.parvum* dataset and obtained $96\%$ accuracy^45^. DDeepDSSR used CNN plus BLSTM-based hybrid network and evaluated the human HS3D dataset, obtaining an accuracy of around $98\%$^34^.

As stated above, various deep learning-based methods have been proposed in the literature. However, users encounter difficulties to choose which deep learning-based method to apply for their data. Therefore, there is a need to compare and evaluate the deep learning-based splice site prediction methods. In order to determine which method might be an appropriate model for splice prediction tasks for a specific dataset, we proposed a framework for experiments to compare the selected promising splice site prediction models such as CNN, BLSTM, and BGRU. The user may see performance variations amongst the splice site prediction models due to the different models and feature learning layers. The evaluated networks use the same optimization method, learning rate, and dense classification layer at the output.

We used accuracy, the F1 score, and A.U.C. Precision-Recall (AUC-PR) as evaluation metrics. We observed that CNN-based networks train orders of magnitude faster than BLSTM-based networks (Fig. 9). To some extent, this might be due to the use of fast convolution computation enabled by cuDNN C used by the TensorFlow library for parallel computations on General Purpose GPUs (GPGPUs), but also, the CNN-based networks have less trainable parameters (and connections) when compared to BLSTM based networks.

Additionally, we suggest that the local correlation in the sequence data is more critical for recognizing their patterns than viewing these sequences as sentences constructed by smaller blocks. This outcome can be explained by the bidirectional characteristics of the DNA and RNA sequences. A language structure presents a clear direction in which the sentences are constructed and meaningful. However, the genomic sequences can be processed from each direction like one-dimensional images with cohesion in small correlated vicinity and depict a complete scene. Therefore, bidirectional LSTM and GRU are preferred because they allow the maintenance of both backward and forward data since they have also been used for splice site prediction^45^.

The accuracy for GRU and LSTM was observed as $55\%$ and $62\%$ as shown in Fig. 4. Results in Fig. 5 showed that bidirectional models outperformed unidirectional models. As genomic sequences are a better fit for bidirectional models using unidirectional networks causes a loss of value. This explains the performance loss observed in our experiments with the unidirectional GRU and LSTM architectures.

There are many deep learning-based splice site predictors in the literature with higher performances as mainly focused on the improving the prediction performances of the networks so that they designed different architectures of deep neural networks. However, this study emphasizes the need for automated evaluation of deep learning models. Unlike other studies, we mainly focused on developing a novel framework for comparing deep learning models for splice site prediction problems rather than building a network with improved accuracy.

Our experiments have shown that the CNN-based model has a better gain than the BLSTM-based model (Fig. 8). CNN-based networks even outperform the BLSTM-based networks with extended training. Besides the feature extraction layers, networks are built as equivalent to each other. So, we conclude that CNN-based networks are more successful in extracting informative features from the sequence, which results in higher classification performance such as accuracy, F1 score, and AUC Precision-Recall.

The CNN-based networks appear to learn the data faster and reach higher accuracy when the network’s complexity increases (Fig. 9). BLSTM-based networks fall behind the CNN-based network in these regards. We observed that convolutional layers in neural networks result in better representations and perform better in the learning process.

We let the BLSTM-based networks train for more epochs after observing that 300 epochs are not enough for these networks to reach their potential. These results are labeled as “extented” in the figures. We concluded that, given enough complexity and time, BLSTM-based network learning performance improved. However, as both models fit the data, CNN-based approaches learn faster and reach a stable level sooner.

Even though collecting and processing the data has been challenging in prior iterations, in the future, these experiments could be conducted with a wide range of sequences to eliminate any effect introduced by the fixed size of the data point. Additionally, the tenfold cross-validation used in this study was challenging and time-consuming since training hundreds of neural networks for an extended time requires considerable resources. Also, both datasets used in this study are composed of canonical splice sites, since we wanted to select similar datasets in terms of sequence length and pattern. Therefore, The only limitation of this study is that our network is not trained to classify non-canonical splice sites.

## Conclusion

This study introduces our deep splice site prediction machine learning framework for multiple machine learning models. We included available deep learning models as building blocks for RNA splice site prediction. To the best of our knowledge, no other work has been developed for evaluating splice detection models to obtain the best possible model in an automated manner. Our framework can help researchers identify the best performing models without laborious training effort to the researcher for an accurate splice site analysis and similar classification tasks. Also, the proposed framework can be used to compare deep learning models with other machine learning tasks.

Our study showed that CNN learns faster than BLSTM and BGRU, and CNN performs better at extracting sequence patterns than BLSTM and BGRU. Since many deep learning-based splice site prediction tools are suggested in the literature, our observations can help to make a selection among CNN or BLSTM, or BGRU-based models for an accurate splice site analysis and similar classification tasks. Also, the proposed blueprint can be used to compare CNN, BLSTM, and BGRU in different problems with different datasets.

Our experiments in this study required long duration preventing experimenting with some parameters. As a future work, we consider adding the feature for experimenting with different hyper-parameter tuning options such as kernel/window size, learning rates, optimizer selections, dropout ratios, and pooling methods.

## Data Availability

The dataset analysed during the current study is available in our GitHub repository: Data Repository.

## Code availability

The code for repeating the experiments are available in our GitHub repository and may be downloaded from following URL address: Code Repository.
